# Maternal autoantibody profiles as biomarkers for ASD and ASD with co-occurring intellectual disability

**DOI:** 10.1038/s41380-022-01633-4

**Published:** 2022-05-26

**Authors:** Alexandra Ramirez-Celis, Lisa A. Croen, Cathleen K. Yoshida, Stacey E. Alexeeff, Joseph Schauer, Robert H. Yolken, Paul Ashwood, Judy Van de Water

**Affiliations:** 1 Department of Internal Medicine, Division of Rheumatology, Allergy, and Clinical Immunology, One Shields Avenue, University of California, Davis, CA 95616 USA; 2 Kaiser Permanente Division of Research, 2000 Broadway, Oakland, CA 94612 USA; 3 Department of Psychiatry and Behavioral Science, Johns Hopkins University School of Medicine, Baltimore, MD USA; 4 UC Davis MIND Institute, 2825 50th St, Sacramento, CA 95817 USA; 5 Department of Medical Microbiology and Immunology, One Shields Avenue, University of California, Davis, CA 95616 USA

**Keywords:** Predictive markers, Neuroscience

## Abstract

Maternal autoantibody-related ASD (MAR ASD) is a subtype of autism in which pathogenic maternal autoantibodies (IgG) cross the placenta, access the developing brain, and cause neurodevelopmental alterations and behaviors associated with autism in the exposed offspring. We previously reported maternal IgG response to eight proteins (CRMP1, CRMP2, GDA LDHA, LDHB, NSE, STIP1, and YBOX) and that reactivity to nine specific combinations of these proteins (MAR ASD patterns) was predictive of ASD risk. The aim of the current study was to validate the previously identified MAR ASD patterns (CRMP1 + GDA, CRMP1 + CRMP2, NSE + STIP1, CRMP2 + STIP1, LDHA + YBOX, LDHB + YBOX, GDA + YBOX, STIP1 + YBOX, and CRMP1 + STIP1) and their accuracy in predicting ASD risk in a prospective cohort employing maternal samples collected prior to parturition. We used prenatal plasma from mothers of autistic children with or without co-occurring intellectual disability (ASD = 540), intellectual disability without autism (ID = 184) and general population controls (GP = 420) collected by the Early Markers for Autism (EMA) study. We found reactivity to one or more of the nine previously identified MAR ASD patterns in 10% of the ASD group compared with 4% of the ID group and 1% of the GP controls (ASD vs GP: Odds Ratio (OR) = 7.81, 95% Confidence Interval (CI) 3.32 to 22.43; ASD vs ID: OR = 2.77, 95% CI (1.19–7.47)) demonstrating that the MAR ASD patterns are strongly associated with the ASD group and could be used to assess ASD risk prior to symptom onset. The pattern most strongly associated with ASD was CRMP1 + CRMP2 and increased the odds for an ASD diagnosis 16-fold (3.32 to >999.99). In addition, we found that several of these specific MAR ASD patterns were strongly associated with ASD with intellectual disability (ASD + ID) and others associated with ASD without ID (ASD-no ID). Prenatal screening for these MAR patterns may lead to earlier identification of ASD and facilitate access to the appropriate early intervention services based on each child’s needs.

## Introduction

Autism spectrum disorder (ASD) is a complex neurodevelopmental disorder characterized by challenges in social communication, restricted interests, and repetitive behaviors [1] that currently affects over 2% of 8-year-olds in the US (1 in 44) [2, 3]. Autism often co-occurs with other conditions such as psychiatric disorders [4, 5] attention deficit hyperactivity disorder (ADHD), intellectual disability (ID), sensory processing, immune dysregulation and gastrointestinal issues [6–8]. Recently, the CDC reported that one-third (34%) of autistic children have intellectual disability (ASD + ID) [3]. It is well recognized that individuals with ASD + ID present significant deficits and challenges with adaptive behavior and therefore have different behavior intervention requirements than those individuals with ASD without ID or ID only [9, 10]. The incidence of autism has increased over the past 50 years; however, we still lack autism-specific biomarkers that would facilitate an earlier diagnosis allowing the provision of directed services based on the ASD sub-phenotypes and associated conditions.

Over the past three decades, multiple studies have suggested an association between maternal immune dysregulation during pregnancy and neurodevelopmental disorders (NDD) in the offspring including autism [11–19]. In particular, gestational exposure to maternal autoantibodies that cross-react with specific fetal brain proteins has been shown to be associated with increased autism risk [20]. We previously reported, in a post-natal sample set from the Childhood Autism Risk- Genes and Environment (CHARGE) study [21] where maternal samples were collected 2–5 years after the birth of the study child, the presence of maternal autoantibodies that recognize eight proteins that are highly expressed in the fetal brain and play significant roles during neurodevelopment. These antigens include collapsin response mediator proteins 1 and 2 (CRMP1, CRMP2), guanine deaminase (GDA), lactate dehydrogenase A and B (LDHA, LDHB), neuron-specific enolase (NSE), stress-induced phosphoprotein-1 (STIP1) and Y-box binding protein 1 (YBOX) [22, 23]. We observed reactivity to single antigens in both the case and control groups; however, reactivity to combinations of two or more specific antigens was associated with autism and was present in up to 18% of the ASD cases; therefore, we termed this subtype of autism “Maternal Autoantibody Related Autism (MAR ASD)” and the patterns that can predict risk as MAR ASD + patterns. The specific patterns contained CRMP1 + GDA, CRMP1 + CRMP2, NSE + STIP1, CRMP2 + STIP1, LDHA + YBOX, LDHB + YBOX, GDA + YBOX, and each of these patterns had 100% positive predictive value (PPV) in their ability to predict ASD. Further, patterns containing STIP1 + YBOX and CRMP1 + STIP1 had 92% and 90% PPV respectively and were present in less than 10% of the typically developing controls [23].

In the current study, we aimed to conduct an external validation of our previous findings by testing the recently validated MAR ASD + patterns and their predictive potential for autism risk using maternal plasma samples from the prospective Early Markers for Autism (EMA) study [24]. The samples studied herein were collected during mid-pregnancy, allowing us to directly evaluate the relationship between gestational exposure to maternal autoantibodies and child neurodevelopmental outcome. We assessed the association between each MAR ASD + pattern and an ASD diagnosis as well as the phenotypic subgroups of ASD as defined by presence or absence of intellectual disability (ID). To evaluate the specificity of the association of these autoantibodies to ASD, we also assessed associations with an outcome of intellectual disability (ID) in the absence of ASD.

## Materials and methods

### Study subjects

The Early Markers for Autism (EMA) study is a population-based, nested case-control study aimed to investigate genetic and immune susceptibility and environmental exposures that contribute to autism risk. The maternal samples were collected between March 2000 and July 2003 from women participating in the prenatal extended α-fetoprotein screening program (XAFP) and included subjects from urban, suburban, and rural areas with multicultural backgrounds in Southern California. Children with ASD or intellectual disability without autism (ID) were ascertained from the California Department of Developmental Services (DDS) that provides services through Regional Centers (RC) to people with autism and other developmental disabilities. All study procedures were approved by the Institutional Review Board at Kaiser Permanente and the State Committee for the Protection of Human Subjects as previously described [24–26].

### Diagnostic verification

The EMA study population and diagnosis classification was recently described in detail by ref. [24]. Briefly, RC records for all children receiving services for ASD or ID were reviewed by expert clinicians and final case status was determined according to the DSM-IV-TR criteria. The ASD group was further categorized into two subgroups based on cognitive scores: Autism without intellectual disability (ASD no-ID) and ASD with intellectual disability (ASD + ID). ID determination was based on RC records with composite scores <70 on all standardized cognitive/functional tests and these designations were reviewed by our expert clinicians. General population (GP) controls were randomly selected from birth certificate files and frequency matched to the ASD group on age, sex, and county of residence at birth. GP controls were not receiving services from a RC and were not verified as typically developing. A summary of the study population can be found in Table 1.

**Table 1:** Group classifications of study population.

Group	Subjects, *N*
Autism spectrum disorder (ASD)	540
Intellectual disability (ID)	184
General Population (GP)	420
ASD with intellectual disability	
Yes (ASD + ID)	285
No (ASD no-ID)	219
Unknown	36

Group classifications included in the Early Markers for Autism (EMA) study.

### Specimen collection

Maternal blood was collected at mid pregnancy (15–20 weeks of gestation) in citrate dextrose. Plasma was separated, labeled, and stored at −80 °C. Prior to use, samples were thawed at room temperature (RT), vortexed, and centrifuged at 13,000 RPM for 10 min.

### Enzyme linked immunosorbent assay (ELISA)

Maternal antibody cross-reactivity against the eight antigens was determined by Enzyme-Linked Immunosorbent Assay (ELISA) using custom- made and commercially available proteins [23, 27]. The protein concentration and plasma dilutions were optimized for each antigen as previously described [23, 27]. In summary, microplates were coated with 100 μl of antigen in carbonate coating buffer pH 9.6, incubated overnight at 4 °C, washed four times with Phosphate Buffered Saline Tween-20 (PBST) 0.05%, and blocked with 2% Super Block (Thermo Scientific, Rockford, lL) for 1 h at RT. Then, 100 µl of the diluted sample was added to each well, incubated for 1.5 h, washed 4 times in PBST 0.05% and incubated for 1 hour with 1:10,000 goat anti-human IgG-HRP IgG (Kirkegaard & Perry Laboratories, Inc., Gaithersburg, MA), followed by four washes with (PBST) 0.05%. We only assessed IgG reactivity as it is the only isotype that can cross the placenta. Finally, 100 µl of BD optEIA liquid substrate for ELISA (BD Biosciences, San Jose, CA) was added to each well, and the reaction was stopped after 4 min with 50 µl of 2 N HCl. The absorbance was measured at 490-450 nm using an iMark Microplate Absorbance Reader (Biorad, Hercules, CA, USA).

### Statistical analysis

For the ELISA, a positive cutoff value for each antigen was determined after plate-plate normalization using an ROC curve, and the Youden’s index as previously described [23, 27]. Comparisons of sociodemographic characteristics between ASD, ID and GP groups were calculated using χ^2^ test (statistical significance threshold set at *p* < 0.05). To evaluate the association between reactivity to each of the MAR ASD + patterns and child outcome, we performed group comparisons using Fisher Exact test (ASD vs GP, ASD vs ID, ID vs GP, and ASD + ID vs ASD no-ID) and calculated the odds ratios (ORs) with 95% confidence intervals (95% CIs) using exact logistic regression, which is appropriate for small or zero cell sizes. Note that a zero-cell size for the number exposed in the reference group results in an unbounded upper confidence interval limit for the OR, displayed as >999.99. Additionally, we used χ^2^ tests (*p* < 0.05) to examine associations between sociodemographic characteristics and the MAR ASD + patterns.

## Results

### Population sociodemographic characteristics

Mothers of autistic children were more likely to be older, non-Hispanic, and have higher education compared with mothers of the ID and GP groups (Table 2). Mothers of ID children were more likely to be younger, less educated, Mexican-born, and deliver prematurely compared with the ASD and GP groups (Table 2). Within the autism group, the mothers of children with ASD + ID were more likely to be multiparous, Hispanic, born in Mexico and have a lower level of education compared with mothers of children with ASD no-ID (Table 3).

**Table 2:** Descriptive characteristics of the EMA study population.

	ASD	ID	GP	ASD vs GP	ASD vs ID	ID vs GP
	*N* = 540	*N* = 184	*N* = 420			
	N (%)	N (%)	N (%)	χ^2^ *p* value	χ^2^ *p* value	χ^2^ *p* value
Maternal age				0.08	**<0.001**	**<0.001**
<20	17 (3.15%)	26 (14.13%)	21 (5.0%)			
20–24	80 (14.81%)	41 (22.28%)	70 (16.67%)			
25–29	146 (27.04%)	51 (27.72%)	129 (30.71%)			
30–34	196 (36.3%)	43 (23.37%)	145 (34.52%)			
>=35	101 (18.7%)	23 (12.5%)	55 (13.1%)			
Maternal race				0.20	**<0.001**	**0.01**
White	399 (73.89%)	160 (86.96%)	334 (79.52%)			
Asian	109 (20.19%)	11 (5.98%)	65 (15.48%)			
Other	27 (5.0%)	12 (6.52%)	19 (4.52%)			
Missing	5 (0.93%)	1 (0.54%)	2 (0.48%)			
Maternal ethnicity				0.14	**<0.001**	**<0.001**
Hispanic	220 (40.74%)	128 (69.57%)	198 (47.14%)			
Not Hispanic	315 (58.33%)	55 (29.89%)	219 (52.14%)			
Missing	5 (0.93%)	1 (0.54%)	3 (0.71%)			
Parity				0.02	**0.005**	0.30
Multiparous	294 (54.44%)	122 (66.3%)	260 (61.9%)			
Primiparious	246 (45.56%)	62 (33.7%)	160 (38.1%)			
Maternal birth country				**0.05**	**<0.001**	**<0.001**
US	267 (49.44%)	83 (45.11%)	203 (48.33%)			
Mexico	130 (24.07%)	84 (45.65%)	127 (30.24%)			
Other	143 (26.48%)	17 (9.24%)	90 (21.43%)			
Maternal education				**0.01**	**<0.001**	**<0.001**
<High School	98 (18.15%)	78 (42.39%)	102 (24.29%)			
High School Grad	114 (21.11%)	47 (25.54%)	112 (26.67%)			
Undergrad College	220 (40.74%)	46 (25.0%)	144 (34.29%)			
Post-Grad College	100 (18.52%)	11 (5.98%)	59 (14.05%)			
Unknown	8 (1.48%)	2 (1.09%)	3 (0.71%)			
Child characteristics						
Child Sex				0.62	**<0.001**	**<0.001**
Male	442 (81.85%)	102 (55.43%)	349 (83.1%)			
Female	98 (18.15%)	82 (44.57%)	71 (16.9%)			
Birth type				0.33	0.93	0.39
Singleton	526 (97.41%)	179 (97.28%)	413 (98.33%)			
Multiple	14 (2.59%)	5 (2.72%)	7 (1.67%)			
Birth weight				0.10	**<0.001**	**<0.001**
<1500 g	5 (0.93%)	14 (7.61%)	6 (1.43%)			
1500–2499 g	37 (6.85%)	25 (13.59%)	16 (3.81%)			
>=2500 g	498 (92.22%)	145 (78.8%)	398 (94.76%)			
Gestational age				0.92	**<0.001**	**<0.001**
<32 weeks	11 (2.04%)	16 (8.7%)	9 (2.14%)			
33–36 weeks	41 (7.59%)	26 (14.13%)	29 (6.9%)			
>=37 weeks	488 (90.37%)	142 (77.17%)	382 (90.95%)			

Demographic differences between groups was calculated by χ^2^ test and *p* values > 0.05 were bolded and considered significant.

*ASD* Autism Spectrum Disorders, *ID* intellectual disability, *GP* general population.

**Table 3:** Descriptive characteristics of ASD phenotypic subgroups.

	ASD + ID	ASD no-ID	ASD-ID vs ASD no-ID
	*N* = 285	*N* = 219	
	*N* (%)	*N* (%)	χ^2^ *p* value
Maternal age			0.18
<20	12 (4.21%)	5 (2.28%)	
20–24	50 (17.54%)	25 (11.42%)	
25–29	71 (24.91%)	67 (30.59%)	
30–34	98 (34.39%)	81 (36.99%)	
>=35	54 (18.95%)	41 (18.72%)	
Maternal race			0.34
White	203 (71.23%)	170 (77.63%)	
Asian	62 (21.75%)	37 (16.89%)	
Other	16 (5.61%)	11 (5.02%)	
Missing	4 (1.4%)	1 (0.46%)	
Maternal ethnicity			**0.03**
Hispanic	129 (45.26%)	77 (35.16%)	
Not Hispanic	152 (53.33%)	141 (64.38%)	
Missing	4 (1.4%)	1 (0.46%)	
Parity			**0.01**
Multiparous	167 (58.6%)	102 (46.58%)	
Primiparious	118 (41.4%)	117 (53.42%)	
Maternal birth country			**0.03**
US	126 (44.21%)	123 (56.16%)	
Mexico	77 (27.02%)	45 (20.55%)	
Other	82 (28.77%)	51 (23.29%)	
Maternal education			**0.00**
<High School	61 (21.4%)	26 (11.87%)	
High School Grad	69 (24.21%)	42 (19.18%)	
Undergrad College	105 (36.84%)	101 (46.12%)	
Post-Grad College	44 (15.44%)	49 (22.37%)	
Unknown	6 (2.11%)	1 (0.46%)	
Child characteristics			
Child sex			0.74
Male	231 (81.05%)	180 (82.19%)	
Female	54 (18.95%)	39 (17.81%)	
Birth type			0.64
Singleton	279 (97.89%)	213 (97.26%)	
Multiple	6 (2.11%)	6 (2.74%)	
Birth weight			0.19
<1500 g	5 (1.75%)	0 (0%)	
1500–2499 g	22 (7.72%)	14 (6.39%)	
>=2500 g	258 (90.53%)	205 (93.61%)	
Gestational age			0.23
<32 weeks	8 (2.81%)	3 (1.37%)	
33–36 weeks	24 (8.42%)	12 (5.48%)	
>=37 weeks	253 (88.77%)	204 (93.15%)	

Demographic differences between groups was calculated by χ^2^ test and *p* values > 0.05 were bolded and considered significant.

*ASD* *+* *ID* Autism spectrum disorder with co-occurring intellectual disability, *ASD no-ID* autism spectrum disorder without co-occurring intellectual disability.

### Autoantibody reactivity against fetal brain antigens

Maternal autoantibody reactivity to at least one antigen was observed in more than half of the study participants in each study group (60% of ASD, 54% of ID, and 57% of GP; χ^2^ *p* = 0.34). Table 4 presents maternal IgG reactivity to the MAR ASD + patterns that predicted ASD risk in our previous discovery study [23] and were validated in this dataset. We found that 10% of the ASD group had significant IgG reactivity to any of the previously identified ASD-specific patterns compared with 4% of the ID group and 1% of GP controls (ASD vs GP: OR = 7.81, 95% CI 3.32–22.43, *p* < 0.001; ASD vs ID: OR = 2.77, 95% CI 1.19–7.47, *p* = 0.01). Although not a statistically significant difference, IgG reactivity to any of the MAR ASD + patterns was nearly 3 times as common among the ID group compared with the GP control group (ID vs GP: OR = 2.72, 95% CI 0.77–9.96, *p* = 0.07).

**Table 4:** Analysis of previously identified maternal antibody patterns (ASD MAR + patterns).

Antibody Pattern	ASD *N* = 540	ID *N* = 184	GP *N* = 420	ASD vs GP	ASD vs GP	ASD vs ID	ASD vs ID	ID vs GP	ID vs GP
	*N* (%)	*N* (%)	*N* (%)	Fisher’s Exact *p* value	OR 95% CI	Fisher’s Exact *p* value	OR 95% CI	Fisher’s Exact *p* value	OR 95% CI
ANY COMBO	55 (10.19)	7 (3.8)	6 (1.43)	**<0.001**	**7.81 (3.32 to 22.43)**	**0.01**	**2.86 (1.27 to 7.6)**	0.07	2.72 (0.77 to 9.96)
CRMP1 + CRMP2	14 (2.59)	0 (0.)	0 (0)	**<0.001**	**15.68 (3.32 to** > **999.99)**	**0.03**	**6.86 (1.45 to** > **999.99)**	NA	NA
CRMP2 + STIP1	9 (1.67)	1 (0.54)	0 (0)	**0.01**	**9.85 (1.99 to** > **999.99)**	0.47	3.1 (0.42 to 136.68)	0.30	2.28 (0.12 to >999.99)
LDHA + YBOX	10 (1.85)	2 (1.09)	1 (0.24)	**0.03**	**7.89 (1.12 to 343.89)**	0.74	1.72 (0.36 to 16.25)	0.22	4.59 (0.24 to 272.28)
GDA + YBOX	6 (1.11)	2 (1.09)	0 (0)	**0.04**	**6.4 (1.21 to** > **999.99)**	1.00	1.02 (0.18 to 10.45)	0.09	5.53 (0.66 to >999.99)
CRMP1 + STIP1	9 (1.67)	0 (0)	1 (0.24)	**0.05**	**7.09 (0.98 to 312.00)**	0.12	4.31 (0.87 to **>**999.99)	1.00	2.28 (0. to 43.37)
NSE + STIP1	5 (0.92)	0 (0)	0 (0)	0.07	5.26 (0.95 to **>**999.99)	0.34	2.30 (0.42 to **>**999.99)	NA	NA
CRMP1 + GDA	8 (1.48)	1 (0.5)	1 (0.24)	0.09	6.29 (0.84 to 280.23)	0.46	2.75 (0.36 to 122.77)	0.52	2.29 (0.03 to 180.15)
LDHB + YBOX	4 (0.74)	0 (0)	0 (0)	0.14	4.13 (0.7 to **>**999.99)	0.58	1.81 (0.31 to >999.99)	NA	NA
STIP1 + YBOX	4 (0.74)	2 (1.09)	3 (0.71)	1.00	1.04 (0.17 to 7.12)	0.65	0.68 (0.10 to 7.57)	0.64	1.53 (0.13 to 13.44)

*ASD* Autism Spectrum Disorders, *ID* intellectual disability, *GP* general population, *CRMP1 and CRMP2* collapsin response mediator proteins 1 and 2, *GDA* guanine deaminase, *NSE* neuron specific enolase, *LDHA-B* lactate dehydrogenase A and B, *STIP1* stress induced phosphoprotein 1, *YBOX* Y-box binding protein 1.

Fisher’s exact test (two-sided) was used to evaluate the association of the patterns with ASD diagnosis and *p* values > 0.05 were bolded and considered significant.

The odds ratios (ORs) with 95% confidence intervals (95% CIs) using exact logistic regression, which is appropriate for small or zero cell sizes. Note that a zero-cell size for the number exposed in the reference group results in an unbounded upper confidence interval limit for the OR, displayed as >999.99.

The pattern with the highest odds associated with ASD was CRMP1 + CRMP2, which was present in ~3% of the ASD group versus 0% of the GP and ID groups. Maternal autoantibody reactivity to both CRMP1 and CRMP2 increased the odds of an ASD diagnosis to nearly 16-fold relative to the GP controls (ASD vs GP: OR = 15.68, 95% CI 3.32->999.99, *p* < 0.001) and over 6-fold relative to the ID group (ASD vs ID: *p* = 0.04, OR = 6.46, 95% CI 1.32->999.99). Other patterns significantly associated with ASD risk included CRMP2 + STIP1, LDHA + YBOX, GDA + YBOX, and CRMP1 + STIP1 when compared to the GP group (Table 4). These patterns did not display reactivity differences between ID and GP, suggesting that reactivity to MAR ASD + patterns is highly correlated with an autism diagnosis.

Further, we evaluated the MAR ASD + patterns for the ASD phenotypic subgroups based on the co-occurrence with ID (Table 5). Reactivity to any of MAR ASD + patterns significantly increased the odds for both ASD + ID (ASD + ID vs GP: OR 8.7, 95% CI 3.52–25.82, *p* = < 0.001,) and ASD no-ID (ASD no-ID vs GP: OR 7.29, 95% CI 2.79–22.44, *p* = < 0.001) with similar OR for both groups. The pattern associated with the highest odds of both ASD + ID and ASD no-ID diagnosis was CRMP1 + CRMP2 (ASD + ID vs GP: OR 18.91, 95% CI 3.81 to >999.99, *p* = <0.001; ASD no-ID vs GP: OR 13.11, 95% CI 2.37 to >999.99, *p* <0.01). LDHA + YBOX pattern also showed similar magnitudes of increased odds of both ASD + ID and ASD no-ID (ASD + ID vs GP: OR 7.46, 95% CI 0.83–354.71, *p* = <0.04 and ASD no-ID vs GP: OR 7.77, 95% CI 0.76–384.79, *p* < 0.05). Although there were no statistically significant differences between ASD + ID vs ASD no-ID with respect to the various antigen patterns, CRMP2 + STIP1 and CRMP1 + GDA trended higher in the ASD + ID group (Table 5). In addition, CRMP2 + STIP1 and CRMP1 + GDA were strongly associated with ASD + ID vs GP but did not reach statistical significance for ASD no-ID vs GP. The CRMP1 + STIP1 and GDA + YBOX patterns were associated with ASD no-ID vs GP but did not reach statistical significance for ASD no-ID vs GP (Table 5).

**Table 5:** Maternal IgG reactivity to MAR ASD + patterns by ASD phenotypic subgroups.

Antibody Pattern	ASD + ID *N* = 285	ASD no-ID *N* = 219	GP *N* = 420	ASD + ID vs GP	ASD + ID vs GP	ASD no-ID vs GP	ASD no-ID vs GP	ASD + ID vs ASD no-ID	ASD + ID vs ASD no-ID
	*N* (%)	*N* (%)	*N* (%)	Fisher’s Exact *p* value	OR 95% CI	Fisher’s Exact *p* value	OR 95% CI	Fisher’s Exact *p* value	OR 95% CI
ANY COMBO	32 (11.23)	21 (9.59)	6 (1.43)	**<0.001**	**8.7 (3.52 to 25.82)**	**< 0.01**	**7.29 (2.79 to 22.44)**	0.66	1.19 (0.64 to 2.25)
CRMP1 + CRMP2	9 (3.16)	5 (2.28)	0 (0)	**<0.001**	**18.91 (3.81 to** > **999.99)**	**< 0.01**	**13.11 (2.37 to** > **999.99)**	0.60	1.39 (0.41 to 5.38)
CRMP2 + STIP1	8 (2.81)	1 (0.46)	0 (0)	**<0.001**	**16.66 (3.3 to** > **999.99)**	0.34	1.92 (0.1 to >999.99)	0.08	6.28 (0.83 to 280.63)
CRMP1 + GDA	7 (2.46)	1 (0.46)	1 (0.24)	**0.01**	**10.52 (1.34 to 476.6)**	1.00	1.92 (0.02 to 151.23)	0.15	5.48 (0.7 to 248.51)
LDHA + YBOX	5 (1.75)	4 (1.83)	1 (0.24)	**0.04**	**7.46 (0.83 to 354.71)**	**0.05**	**7.77 (0.76 to 384.79)**	1.00	0.96 (0.2 to 4.9)
NSE + STIP1	2 (0.7)	2 (0.91)	0 (0.)	0.16	3.57 (0.42 to >999.99)	0.12	4.65 (0.55 to >999.99)	1.00	0.77 (0.06 to 10.66)
LDHB + YBOX	2 (0.7)	2 (0.91)	0 (0.)	0.16	3.57 (0.42 to >999.99)	0.12	4.65 (0.55 to >999.99)	1.00	0.77 (0.06 to 10.66)
CRMP1 + STIP1	4 (1.4)	5 (2.28)	1 (0.24)	0.16	5.95 (0.58 to 294.42)	**0.02**	**9.76 (1.08 to 464.1)**	0.51	0.61 (0.12 to 2.87)
GDA + YBOX	1 (0.35)	4 (1.83)	0 (0)	0.40	1.47 (0.08 to >999.99)	**0.01**	**10.26 (1.73 to** > **999.99)**	0.17	0.19 (0.02 to 1.94)
STIP1 + YBOX	1 (0.35)	2 (0.91)	3 (0.71)	0.65	0.49 (0.01 to 6.14)	1.00	1.28 (0.11 to 11.27)	0.58	0.38 (0.01 to 7.4)

*ASD* *+* *ID* Autism spectrum disorder with co-occurring intellectual disability, *ASD no-ID* autism spectrum disorder without co-occurring intellectual disability, *GP* general population, *CRMP1 and CRMP2* collapsin response mediator proteins 1 and 2, *GDA* guanine deaminase, *NSE* neuron specific enolase, *LDHA-B* lactate dehydrogenase A and B, *STIP1* stress induced phosphoprotein 1, *YBOX* Y-box binding protein 1.

Fisher’s exact test (two-sided) was used to evaluate the association of the patterns with ASD diagnosis and *p* values > 0.05 were bolded and considered significant.

The odds ratios (ORs) with 95% confidence intervals (95% CIs) using exact logistic regression, which is appropriate for small or zero cell sizes. Note that a zero-cell size for the number exposed in the reference group results in an unbounded upper confidence interval limit for the OR, displayed as >999.99.

Lastly, we explored if having an ASD MAR + pattern was associated with any sociodemographic factors (Supplementary fig. 1). There were no statistically significant differences in sociodemographic profiles between women with reactivity to any of the ASD-specific antigen combination and women with no reactivity to any combination. While some patterns were present only in Hispanic women (NSE + STIP1) or women who delivered male offspring (LDHA + YBOX and GDA + YBOX), the study was underpowered for statistical analysis of individual patterns.

## Discussion

During pregnancy, the body goes through numerous adaptations and changes [28] including the establishment of maternal-fetal immune homeostasis, which provides protection against pathogens while allowing the allogenic embryo to implant and develop [29, 30]. Among the five primary immunoglobulin isotypes, IgG, IgM, IgA, IgD, and IgE, only maternal IgG can cross the uninflamed placenta in appreciable quantities and provide passive protection to the fetus beginning in gestational week 14 [31]. However, pathogenic autoantibodies can also cross the placenta to react with antigens in the fetal compartment, and may contribute to neonatal diseases such as neonatal lupus, neonatal anemia, neonatal pemphigus and neonatal myasthenia gravis [32] as well as neurodevelopmental disorders such as ID [33–35], ADHD [36], and ASD [15, 22, 23, 37]. This project aimed to expand our earlier discovery results and evaluate the previously described MAR ASD + patterns [23] as potential early biomarkers for ASD using maternal blood samples collected mid-gestation [24], allowing us to assess the pathological significance of gestational exposure to these maternal autoantibodies to child neurodevelopmental outcome.

Over half of the maternal samples in each study group had IgG reactivity to one or more antigens, indicating that reactivity to an individual protein-target is not associated with child outcome. However, reactivity to any of the previously described MAR ASD + patterns, which occurred in 10% of the ASD group and 1% of the GP group, was highly predictive of ASD risk, consistent with our recent reports [22, 23]. Of interest, 4% of the ID group also reacted to one or more of the patterns but this was not significantly different than the 1% among the GP population, suggesting that MAR ASD + patterns were more strongly associated with an ASD diagnosis. Consistent with our prior study that analyzed maternal samples collected 2–5 years after the birth of the study child, in the present study, maternal IgG reactivity during pregnancy to CRMP1 + CRMP2, CRMP2 + STIP1, LDHA + YBOX, GDA + YBOX, and CRMP1 + STIP1 significantly increased the odds of an ASD diagnosis in the exposed child. However, the MAR ASD + patterns found most frequently among the ASD group differed between the two studies. The most abundant patterns in our previous study using the CHARGE study samples [23], were CRMP1 + GDA, followed by CRMP1 + CRMP2, and NSE + STIP1. In the present study (EMA), CRMP1 + GDA and NSE + STIP1 were less prevalent and only trended towards significantly increasing the odds for ASD diagnosis. The distribution discrepancies between the CHARGE and EMA studies could be due to demographic or geographical differences of the study populations (Northern CA vs Southern CA), the years during which the pregnancies occurred (2002–2012 vs 2000–2003), and/or the time period during which the maternal samples were collected (2–5 years post-delivery vs. second trimester) [21, 24].

In the current study, the MAR ASD + pattern associated with the highest odds of ASD was CRMP1 + CRMP2, increasing odds of ASD nearly 16-fold compared to the GP controls. This pattern was associated with the highest odds for both ASD + ID and ASD no-ID compared with GP controls. In contrast, the patterns CRMP2 + STIP1 and CRMP1 + GDA increased the odds for an ASD + ID diagnosis suggesting that maternal autoantibodies against these protein combinations could target shared pathways between ASD and ID, altering both behavior and cognition. Interestingly, GDA + YBOX and CRMP1 + STIP1 increased the odds of the ASD no-ID phenotype. Each of the target proteins are biologically relevant due to their key role in brain development as dendritic arborization, organization and maintenance of neural network [38–41], brain metabolism [39], cognition/memory formation [42], neuroprotection, and CNS homeostasis [43, 44]. Previous studies have shown that mutations or deficits in these proteins are associated with neurological pathology, such as ASD [45, 46], schizophrenia [47, 48], ADHD [49, 50], and intellectual disability [51, 52]. Therefore, we hypothesize that gestational exposure to maternal autoantibodies that cross-react with relevant brain proteins could have an additive effect in altering neurodevelopmental pathways and contribute to ASD etiology and pathogenesis. Thus the different MAR ASD + patterns presented here could serve as biomarkers not only for ASD but for specific ASD phenotypes.

Other studies have looked at maternal IgG reactivity to single proteins and their association with NDD, reviewed in [15, 16, 20]. For example, other clinical studies and animal models have reported that maternal antibodies that target CASPR2, a potential ASD-risk biomarker, alter brain anatomy, function, and are related to autism manifestations and learning issues in the exposed offspring [20, 35, 37, 53–55]. Using gestational plasma collected from a subset of a large Danish study with over 100,000 participants, Coutinho and collaborators investigated maternal autoantibody against multiple brain proteins (including NMDA and CASPR2) and their association with child outcome. They reported a strong association between autoantibody reactivity to NMDA and CASPR2 and ID, but not ASD [35]. Thus, the utility of using maternal reactivity to NMDA and CASPR2 antibodies as biomarkers of ID and ASD would need clinical additional validation.

Although ASD can be diagnosed as early as 18 months of age, most children receive a diagnosis after the three years of age [56] thereby delaying early intervention services that would improve life outcomes. There is a high co-occurrence of ASD with intellectual disability (30–70%) [3, 57], and the ability to distinguish between ID, ASD no-ID and ASD + ID in the first years of life would enable clinicians to provide more targeted behavioral interventions [10]. Therefore, it is of clinical importance to develop biomarkers that can not only identify risk of ASD, but provide information regarding the ASD phenotype, allowing the clinical intervention strategy to be better tailored to the child’s specific needs and strengths [10]. The MAR ASD + patterns presented herein provide information regarding potential candidates for use as biomarkers of ASD risk to be further validated in future studies.

Some limitations of the current study deserve mention. First, we could not verify that all children in the GP control group were typically developing. While the GP controls had never been clients of a Regional Center, it is possible that some may have an undiagnosed developmental disorder, which could account for the presence of some of the MAR ASD + patterns in 1% of the GP group. A second limitation was the small number of maternal samples with MAR ASD + patterns in the ASD + ID and ASD no-ID groups. This reduced our ability to reach statistical significance for some of the less common patterns. Future studies are underway to expand the study population and include information about environmental factors which might increase the level of autoantibodies such as infection, medications, cigarette smoking, and gestational exposure to wildfires and farmland pesticides.

One of the greatest strengths of the current study is that the samples were collected during mid-pregnancy, demonstrating the predicative value of maternal IgG reactivity against MAR ASD + patterns and child outcomes. In addition, the EMA study included children with ASD no-ID and ASD + ID as well as children with other developmental disorders without ASD, allowing us to identify maternal autoantibody patterns predictive of specific neurodevelopmental phenotypes. Finally, we had relevant sociodemographic information for this diverse sample set that allowed us to make interesting observations that should be confirmed in for future studies. Forthcoming research will include clinical validation of the MAR ASD + patterns in larger cohorts from different geographical regions, and the creation of in vitro and in vivo models to study the biological pathways involved in MAR ASD while considering ASD in co-occurrence with ID. We aim to develop accurate biomarkers to provide clinicians with additional tools for an earlier diagnosis of ASD, and to better tailor intervention services based on the ASD phenotype and the child’s individual strengths and specific challenges.

## Supplementary information

Supplementary Table 1
