# Supplementary material for: Maternal autoantibody profiles as biomarkers for ASD and ASD with co-occurring intellectual disability
Source: Mol Psychiatry. 2022 May 26;27(9):3760–7. doi: 10.1038/s41380-022-01633-4 (PMC9708563; doi:10.1038/s41380-022-01633-4)
Supplement: Supplementary file 1 — Supplementary Table 1 [file 41380_2022_1633_MOESM1_ESM.docx]

Supplementary Table 1. Subject demographics associated with each MAR pattern.

|  | **CRMP1+GDA** | **CRMP1+CRMP2** | **NSE+STIP1** | **CRMP2+STIP1** | **LDHA+YBOX** | **LDHB+YBOX** | **GDA+YBOX** | **STIP1+YBOX** | **CRMP1+STIP1** | **Any MAR COMBO** |
| --- | --- | --- | --- | --- | --- | --- | --- | --- | --- | --- |
|  | **N=10** | **N=14** | **N=5** | **N=10** | **N=13** | **N=4** | **N=8** | **N=9** | **N=10** | **χ2 pval** |
| **Maternal Characteristics** |  |  |  |  |  |  |  |  |  |  |
| Maternal Age |  |  |  |  |  |  |  |  |  | 0.86 |
| <20 | 0 (0.%) | 0 (0.%) | 0 (0.%) | 0 (0.%) | 1 (7.69%) | 0 (0.%) | 1 (12.5%) | 1 (11.11%) | 0 (0.%) |  |
| 20-24 | 4 (40.%) | 2 (14.29%) | 1 (20.%) | 1 (10.%) | 3 (23.08%) | 1 (25.%) | 2 (25.%) | 2 (22.22%) | 2 (20.%) |  |
| 25-29 | 1 (10.%) | 3 (21.43%) | 0 (0.%) | 3 (30.%) | 4 (30.77%) | 1 (25.%) | 2 (25.%) | 3 (33.33%) | 3 (30.%) |  |
| 30-34 | 5 (50.%) | 6 (42.86%) | 3 (60.%) | 4 (40.%) | 2 (15.38%) | 1 (25.%) | 1 (12.5%) | 2 (22.22%) | 4 (40.%) |  |
| >=35 | 0 (0.%) | 3 (21.43%) | 1 (20.%) | 2 (20.%) | 3 (23.08%) | 1 (25.%) | 2 (25.%) | 1 (11.11%) | 1 (10.%) |  |
| Maternal Race |  |  |  |  |  |  |  |  |  | 0.48 |
| White | 7 (70.%) | 9 (64.29%) | 2 (40.%) | 6 (60.%) | 12 (92.31%) | 4 (100.%) | 6 (75.%) | 9 (100.%) | 5 (50.%) |  |
| Asian | 1 (10.%) | 1 (7.14%) | 1 (20.%) | 0 (0.%) | 0 (0.%) | 0 (0.%) | 1 (12.5%) | 0 (0.%) | 0 (0.%) |  |
| Other | 2 (20.%) | 4 (28.57%) | 2 (40.%) | 4 (40.%) | 1 (7.69%) | 0 (0.%) | 1 (12.5%) | 0 (0.%) | 5 (50.%) |  |
| Missing | 0 (0.%) | 0 (0.%) | 0 (0.%) | 0 (0.%) | 0 (0.%) | 0 (0.%) | 0 (0.%) | 0 (0.%) | 0 (0.%) |  |
| Maternal Ethnicity |  |  |  |  |  |  |  |  |  | 0.49 |
| Hispanic | 6 (60.%) | 8 (57.14%) | 5 (100.%) | 4 (40.%) | 6 (46.15%) | 1 (25.%) | 5 (62.5%) | 5 (55.56%) | 6 (60.%) |  |
| Not Hispanic | 4 (40.%) | 6 (42.86%) | 0 (0.%) | 6 (60.%) | 7 (53.85%) | 3 (75.%) | 3 (37.5%) | 4 (44.44%) | 4 (40.%) |  |
| Missing | 0 (0.%) | 0 (0.%) | 0 (0.%) | 0 (0.%) | 0 (0.%) | 0 (0.%) | 0 (0.%) | 0 (0.%) | 0 (0.%) |  |
| Parity |  |  |  |  |  |  |  |  |  | 0.76 |
| Primiparous | 2 (20.%) | 6 (42.86%) | 3 (60.%) | 3 (30.%) | 7 (53.85%) | 1 (25.%) | 4 (50.%) | 4 (44.44%) | 5 (50.%) |  |
| Multiparous | 8 (80.%) | 8 (57.14%) | 2 (40.%) | 7 (70.%) | 6 (46.15%) | 3 (75.%) | 4 (50.%) | 5 (55.56%) | 5 (50.%) |  |
| Maternal Birth Country |  |  |  |  |  |  |  |  |  | 0.82 |
| US | 4 (40.%) | 5 (35.71%) | 4 (80.%) | 2 (20.%) | 10 (76.92%) | 2 (50.%) | 4 (50.%) | 8 (88.89%) | 1 (10.%) |  |
| Mexico | 3 (30.%) | 5 (35.71%) | 1 (20.%) | 4 (40.%) | 1 (7.69%) | 0 (0.%) | 1 (12.5%) | 0 (0.%) | 5 (50.%) |  |
| Other | 3 (30.%) | 4 (28.57%) | 0 (0.%) | 4 (40.%) | 2 (15.38%) | 2 (50.%) | 3 (37.5%) | 1 (11.11%) | 4 (40.%) |  |
| Maternal Education |  |  |  |  |  |  |  |  |  | 0.70 |
| < High School | 3 (30.%) | 2 (14.29%) | 0 (0.%) | 4 (40.%) | 1 (7.69%) | 1 (25.%) | 2 (25.%) | 2 (22.22%) | 1 (10.%) |  |
| High School Grad | 4 (40.%) | 3 (21.43%) | 1 (20.%) | 4 (40.%) | 8 (61.54%) | 3 (75.%) | 1 (12.5%) | 2 (22.22%) | 3 (30.%) |  |
| Undergrad College | 3 (30.%) | 5 (35.71%) | 3 (60.%) | 0 (0.%) | 2 (15.38%) | 0 (0.%) | 2 (25.%) | 2 (22.22%) | 4 (40.%) |  |
| Post-Grad College | 0 (0.%) | 4 (28.57%) | 1 (20.%) | 2 (20.%) | 2 (15.38%) | 0 (0.%) | 3 (37.5%) | 2 (22.22%) | 2 (20.%) |  |
| Unknown | 0 (0.%) | 0 (0.%) | 0 (0.%) | 0 (0.%) | 0 (0.%) | 0 (0.%) | 0 (0.%) | 1 (11.11%) | 0 (0.%) |  |
| **Child Characteristics** |  |  |  |  |  |  |  |  |  |  |
| Child Sex (χ2 pval) | 0.54 | 0.96 | 0.92 | 0.17 | 0.05 | 0.88 | 0.13 | 0.41 | 0.88 | 0.38 |
| Male | 7 (70.%) | 11 (78.57%) | 4 (80.%) | 6 (60.%) | 13 (100.%) | 3 (75.%) | 8 (100.%) | 6 (66.67%) | 8 (80.%) |  |
| Female | 3 (30.%) | 3 (21.43%) | 1 (20.%) | 4 (40.%) | 0 (0.%) | 1 (25.%) | 0 (0.%) | 3 (33.33%) | 2 (20.%) |  |
| Birth Type |  |  |  |  |  |  |  |  |  | 0.86 |
| Singleton | 10 (100.%) | 13 (92.86%) | 5 (100.%) | 10 (100.%) | 13 (100.%) | 4 (100.%) | 8 (100.%) | 9 (100.%) | 9 (90.%) |  |
| Multiple | 0 (0.%) | 1 (7.14%) | 0 (0.%) | 0 (0.%) | 0 (0.%) | 0 (0.%) | 0 (0.%) | 0 (0.%) | 1 (10.%) |  |
| Birth Weight |  |  |  |  |  |  |  |  |  | 0.44 |
| <1500 g | 0 (0.%) | 0 (0.%) | 0 (0.%) | 0 (0.%) | 0 (0.%) | 0 (0.%) | 0 (0.%) | 0 (0.%) | 0 (0.%) |  |
| 1500-2499 g | 0 (0.%) | 2 (14.29%) | 1 (20.%) | 0 (0.%) | 1 (7.69%) | 0 (0.%) | 1 (12.5%) | 0 (0.%) | 0 (0.%) |  |
| >=2500 g | 10 (100.%) | 12 (85.71%) | 4 (80.%) | 10 (100.%) | 12 (92.31%) | 4 (100.%) | 7 (87.5%) | 9 (100.%) | 10 (100.%) |  |
| Gestational Age |  |  |  |  |  |  |  |  |  | 0.67 |
| <32 weeks | 0 (0.%) | 0 (0.%) | 0 (0.%) | 0 (0.%) | 0 (0.%) | 0 (0.%) | 1 (12.5%) | 0 (0.%) | 0 (0.%) |  |
| 33-36 weeks | 0 (0.%) | 2 (14.29%) | 0 (0.%) | 0 (0.%) | 2 (15.38%) | 1 (25.%) | 0 (0.%) | 0 (0.%) | 1 (10.%) |  |
| >=37 weeks | 10 (100.%) | 12 (85.71%) | 5 (100.%) | 10 (100.%) | 11 (84.62%) | 3 (75.%) | 7 (87.5%) | 9 (100.%) | 9 (90.%) |  |

Abbreviations: CRMP1 and CRMP2, collapsin response mediator proteins 1 and 2; GDA, guanine deaminase; NSE, neuron specific enolase; LDHA-B, lactate dehydrogenase A and B; STIP1, stress induced phosphoprotein 1 and YBOX, Y-box binding protein 1. Demographic differences between groups was calculated by χ^2^ test and p-values > 0.05 were considered significant.
